# Phytochemical Profiling and Molecular Insights of *Centaurea lycaonica*: Apoptosis Induction via the Intrinsic Pathway in Endometrial Cancer Cells

**DOI:** 10.3390/ph18101558

**Published:** 2025-10-16

**Authors:** Ayşe Kübra Karaboğa Arslan, Rümeysa Korubaşı, Leyla Paşayeva, Nuh Mehmet Bozkurt, Osman Tugay

**Affiliations:** 1 Department of Pharmacology, Faculty of Pharmacy, Erciyes University, Kayseri 38039, Türkiye; 4025330011@erciyes.edu.tr (R.K.); mehmetbozkurt@erciyes.edu.tr (N.M.B.); 2 Department of Pharmacognosy, Faculty of Pharmacy, Erciyes University, Kayseri 38039, Türkiye; leylapasayeva@erciyes.edu.tr; 3 Department of Pharmaceutical Botany, Faculty of Pharmacy, Selcuk University, Konya 42130, Türkiye; otugay@selcuk.edu.tr

**Keywords:** Bax, caspase 3, *C. lycaonica*, LC-HRMS, RL95-2

## Abstract

**Background/Objectives**: The *Centaurea* genus is characterized by many species, a broad biological diversity, and a rich secondary metabolite content. These species exhibit various biological activities, including antioxidant, anti-inflammatory, antimicrobial, antiproliferative, and wound-healing properties. However, there are limited anticancer research studies available on the species. This study aims to investigate the potential cytotoxic effects of dichloromethane (CRD) and methanol (CRM) extracts obtained from the root of the endemic *Centaurea lycaonica* to clarify the mechanism of apoptosis by the intrinsic pathway on the human endometrial cancer cell line RL95-2 based on phytochemical analysis. **Methods**: The cytotoxicity studies were performed using a Real-Time Cell Analyzer (xCELLigence) and the MTT assay. The activities of caspase 3, caspase 9, Bax, and Bcl-2 were evaluated to investigate the molecular mechanism of apoptosis. LC-HRMS determined the phytochemical content of extracts. **Results**: CRD and CRM had a concentration-dependent effect in increasing caspase 3 and 9 activities and Bax/Bcl-2 ratios compared to the control with low IC_50_ values. **Conclusions**: Apoptosis induction was more pronounced with CRM, which was enriched in hesperidin; this association warrants targeted validation with purified standards.

## 1. Introduction

Endometrial cancer is one of the most common and life-threatening types of cancer in women. According to Global Cancer 2022 data, the worldwide incidence and mortality rates of endometrial cancer are 8.4% and 1.7%, respectively. In Turkey, the incidence rate is 14.3%, while the mortality rate is 2.4% [1]. The primary treatments for endometrial cancer include surgery, radiotherapy, chemotherapy, and combinations of these modalities. Cisplatin and doxorubicin (Dox) have been shown to increase survival rates [2].

**Figure 1:**
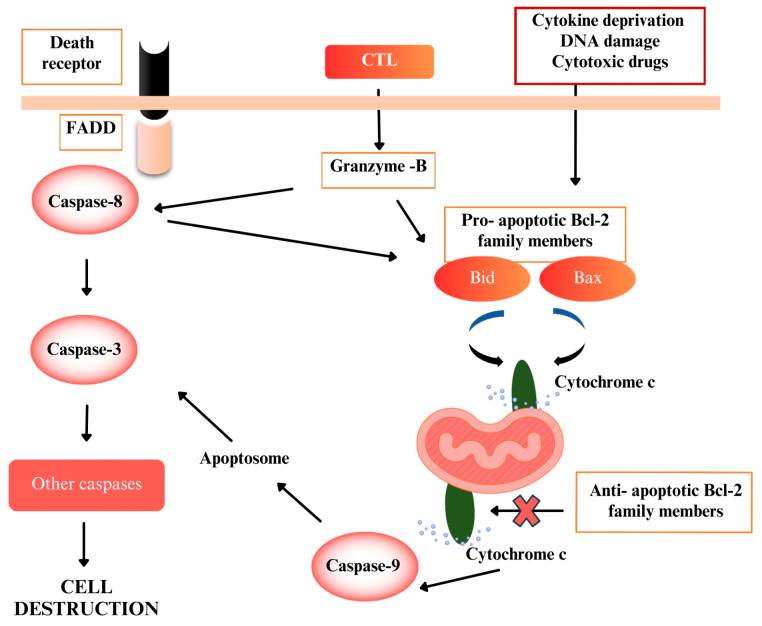
Pathways that activate apoptosis (FADD: Fas-associated death protein; CTL: Cytotoxic T lymphocytes).

The suppression of apoptosis is a fundamental mechanism that enables cancer cell survival and accumulation. Consequently, the therapeutic targeting of apoptotic pathways has become a cornerstone strategy for cancer treatment [3]. Caspases, which play a role in the regulation of apoptosis, are cysteine proteases in the cell in an inactive form and activate each other through a cascade of reactions [4]. The Bcl-2 family, which includes the most crucial apoptosis genes, consists of both pro-apoptotic and anti-apoptotic members with opposing effects [5]. The mechanism by which caspases and Bcl-2 family members activate apoptosis is illustrated in Figure 1.

Death receptors on the cell membrane, such as Fas-associated death domain protein (FADD), lead to the aggregation of caspase-8. Activated caspase-8 subsequently activates caspase-3, initiating a cascade reaction that activates other caspases, ultimately leading to cellular degradation. Caspase-8 also facilitates the activation of the pro-apoptotic protein Bid. Bid, which alters mitochondrial membrane potential, induces the activation of Bax and Bak, promoting the release of cytochrome c into the cytosol. Caspase-9, activated by cytochrome c, forms the apoptosome, which activates caspase-3, triggering the activation of additional caspase groups.

Granzyme B, a caspase-like serine protease secreted by cytotoxic T lymphocytes (CTLs), promotes apoptosis by directly activating caspase-8 and Bid, initiating critical apoptotic events. Additionally, cytokine deprivation, DNA damage, and cytotoxic drugs can directly stimulate apoptotic genes of the Bcl-2 family to induce apoptosis [6].

Targeted cancer research is being conducted to elucidate endometrial cancer development mechanisms and explore new treatment options. There are studies in the literature that show the anticancer activity of *Centaurea* species by enhancing apoptosis through caspase 3/9 and Bax/Bcl-2 pathways [7,8,9].

**Figure 2:**
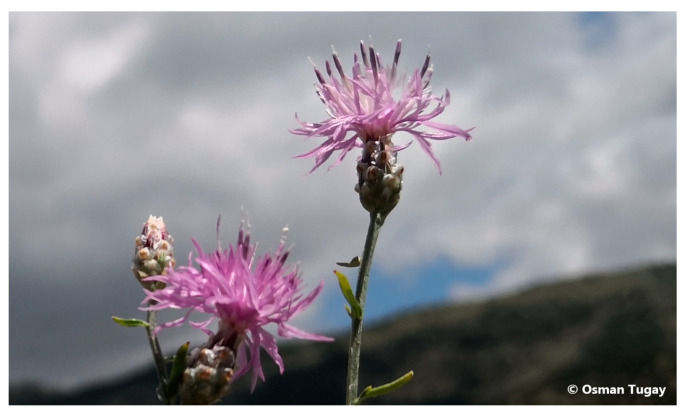
*C. lycaonica* Boiss. & Heldr. (Photo: Prof. Dr. Osman Tugay).

*Centaurea* L., one of the largest genera in the Asteraceae family, is an herbaceous, annual, or perennial flowering plant genus. This genus, which is found in Western Asia, the Northern Hemisphere, Central Europe, and the Mediterranean Basin, includes 172 species in Turkey, with an endemism rate of 64% [10,11,12]. Studies have demonstrated the antioxidant [13], anti-inflammatory [14], antibacterial [15], antimicrobial [16], and antiproliferative [17] activities of plants belonging to these species. Additionally, research suggests that their antioxidant capacity and fatty acid content make them a potential food source [18,19]. *C. lycaonica* (Figure 2) is an endemic species of the *Centaurea* genus, growing only in the Konya region of Turkey. Although there are limited studies on this species in the literature, its high flavonoid content and significant levels of phenolic compounds expressed as gallic acid equivalents suggest its potential as an antioxidant agent [20].

Recent in vitro cytotoxic and apoptotic studies on *Centaurea* species have evaluated the effects of these species on cervical [8], ovarian [13], colon [21], and breast [22] cancer cell lines, reporting promising results. Moreover, in various studies, *Centaurea* species extracts and compounds isolated from these plants, with their rich secondary metabolite content, have demonstrated anticancer activity [23,24,25].

In our previous study published in the literature, the root of *C. lycaonica* was shown to have potential anticancer activity in the HeLa human cervical cancer cell line [26]. In another study of ours, the apoptotic and cytotoxic activities of the aerial parts of *C. lycaonica* were elucidated on the HeLa cell line [27]. Aside from our study, no research has investigated the molecular mechanism underlying the anticancer activity of the *C. lycaonica* species on endometrial cancer. This study investigates the potential effects of dichloromethane (CRD) and methanol (CRM) extracts obtained from the root of *C. lycaonica* to clarify the apoptotic effects on human endometrial cancer cells. Also, the differences between CRD and CRM regarding the biological activity of endometrial cancer cells have been identified based on phytochemical analysis. The present study is significant as it aims to fill this gap in the literature by investigating the potential effects of *C. lycaonica* on endometrial cancer.

## 2. Results

### 2.1. LC-HRMS Results

#### 2.1.1. Qualitative Analyses Results 

**Figure 3:**
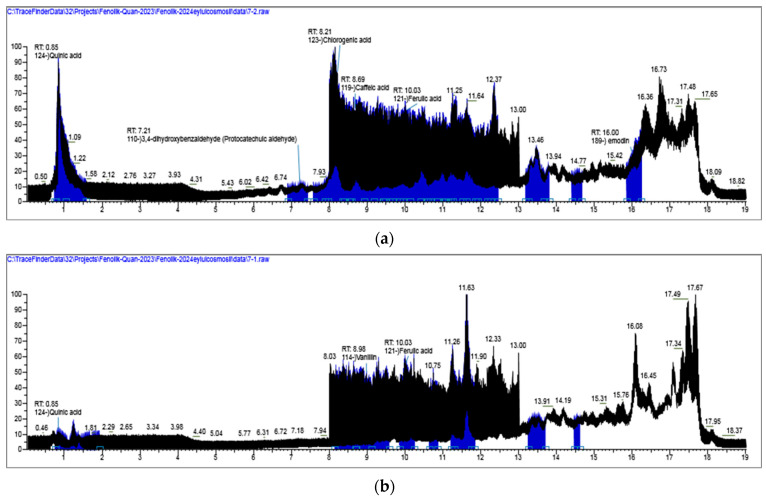
(**a**) TIC profile of standard compounds and CRM extract.; (**b**) TIC profile of standard compounds and CRD extract. (The black chromatogram: the total ion chromatogram (TIC) of the standard compound mixture. The blue chromatogram: the total ion chromatogram (TIC) of the CHM extract).

Our previous phytochemical investigations on *Centaureae* and related taxa provided a solid basis for anticipating the presence of specific phenolic constituents in the studied plant material [20]. The chemical composition of the CRD and CRM extracts was characterized based on accurate mass measurements, fragmentation patterns, and comparison with retention time. Both positive- and negative-ionization modes were tested, but the negative-ion mode proved to be more suitable due to its higher sensitivity, simpler fragmentation pathways, and reduced baseline noise. The total ion chromatograms (TIC) are provided in Figure 3. As listed in Table 1, the extracts of CRM and CRD were found to contain 10 phenolic acids (4-hydroxybenzoic acid, salicylic acid, syringic acid, vanillic acid, coumaric acid, caffeic acid, ferulic acid, sinapic acid, chlorogenic acid, 3-(4-hydroxyphenyl) propionic acid), 9 flavonoids (apigenin, apigenin 7-glucuronide, rutin, luteolFin-7-O-glucuronide, diosmetin, myricetin, orientin, afzelin, hesperidin), 2 phenolic aldehyde (3,4-dihydroxybenzaldehyde and vanilin), 1 organic acid (quinic acid), 1 tannin (ellagic acid).

#### 2.1.2. Quantative Analyses Results

Linearity of the methods was established by triplicate injections of each concentration of standard solutions. The response function of the standards calibration curve was described in Figure S2. The quantitative results of compounds are given in Table 1. Quantitative profiling indicated that the CRM extract was particularly rich in hesperidin (48,503.884 µg/g extract (dw)), rutin (3639.834 µg/g extract (dw)), and chlorogenic acid (2866.98 µg/g extract (dw)). In contrast, diosmetin (216.414 µg/g dw)), vanillin (177.19 µg/g extract (dw)), and ferulic acid (150.306 µg/g extract (dw)) were the main constituents of the CRD extract. As a result of LC-MS/MS analysis for hesperidin, a flavanone glycoside, exhibited a prominent deprotonated molecular ion [M − H]^−^ at *m*/*z* 609 in negative ionization mode. The MS/MS fragmentation of this precursor ion revealed characteristic product ions that are consistent with the loss of the rutinose moiety and subsequent fragmentation of the aglycone hesperetin. The most intense fragment ion was observed at *m*/*z* 301, corresponding to the hesperetin aglycone formed by cleavage of the glycosidic bond (Figure S1) [28].

It is noteworthy that the principal compounds of CRD were also present in CRM at lower concentrations, while hesperidin, rutin, and chlorogenic acid were more prominent in CRM. Overall, these results indicate that the biological effects of the extracts are likely related to their dominant metabolites.

### 2.2. Effects of the C. lycaonica Extracts on the Cell Viability

**Figure 4:**
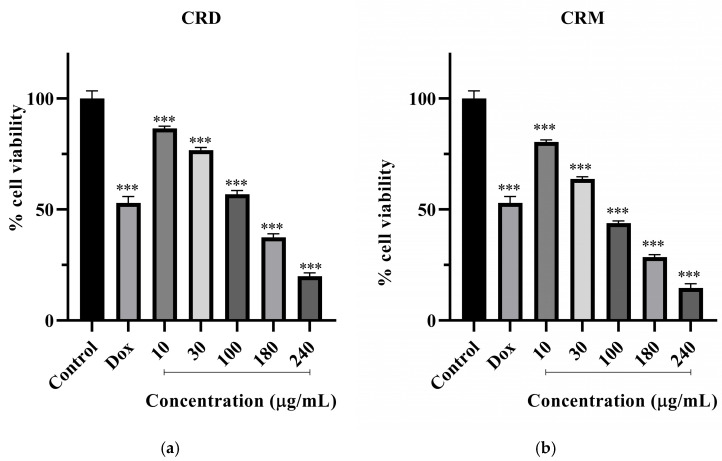
Cell viability of (**a**) CRD and (**b**) CRM on RL95-2 for 48 h (Data were analyzed using One-way ANOVA and post hoc Dunnett’s test (for comparison with the control group) in GraphPad Prism 8.3.0. All groups are presented as percentage changes relative to the untreated control. Results are presented as mean ± SD (*n* = 3). The *p*-value was <0.05. Significance levels: *** < 0.001. CRD: *C. lycaonica* root dichloromethane extract, CRM: *C. lycaonica* root methanol extract, Dox: Doxorubicin (3.37 µg/mL).

The percentage of cell viability in the RL95-2 cell line after treatment with CRD and CRM at concentrations of 10, 30, 100, 180, and 240 µg/mL is presented in Figure 4a,b. The Dox IC_50_ value was 3.37 µg/mL for 48 h. and was used as a positive control. According to the values shown in the figure, the MTT assay results demonstrated that CRD and CRM significantly reduced cell viability in a concentration-dependent manner on RL95-2 cells at all concentrations (*p* < 0.001). At a 30 µg/mL concentration, CRD reduced cell viability to 76.79% (*p* < 0.001), while CRM reduced it to 63.75% (*p* < 0.001). At a concentration of 100, 180, and 240 µg/mL, CRD reduced cell viability to 56.87% (*p* < 0.001), 37.40% (*p* < 0.001), and 19.49% (*p* < 0.001), respectively. At concentrations of 100 and 180 µg/mL, CRM reduced cell viability to between 25% (*p* < 0.001) and 45% (*p* < 0.001). At the highest concentration of 240 µg/mL, CRM exhibited the most significant activity, reducing cell viability to 14.66% (*p* < 0.001).

### 2.3. Monitoring of Cytotoxicity of C. lycaonica Extracts in Real-Time Using xCELLigence System

**Figure 5:**
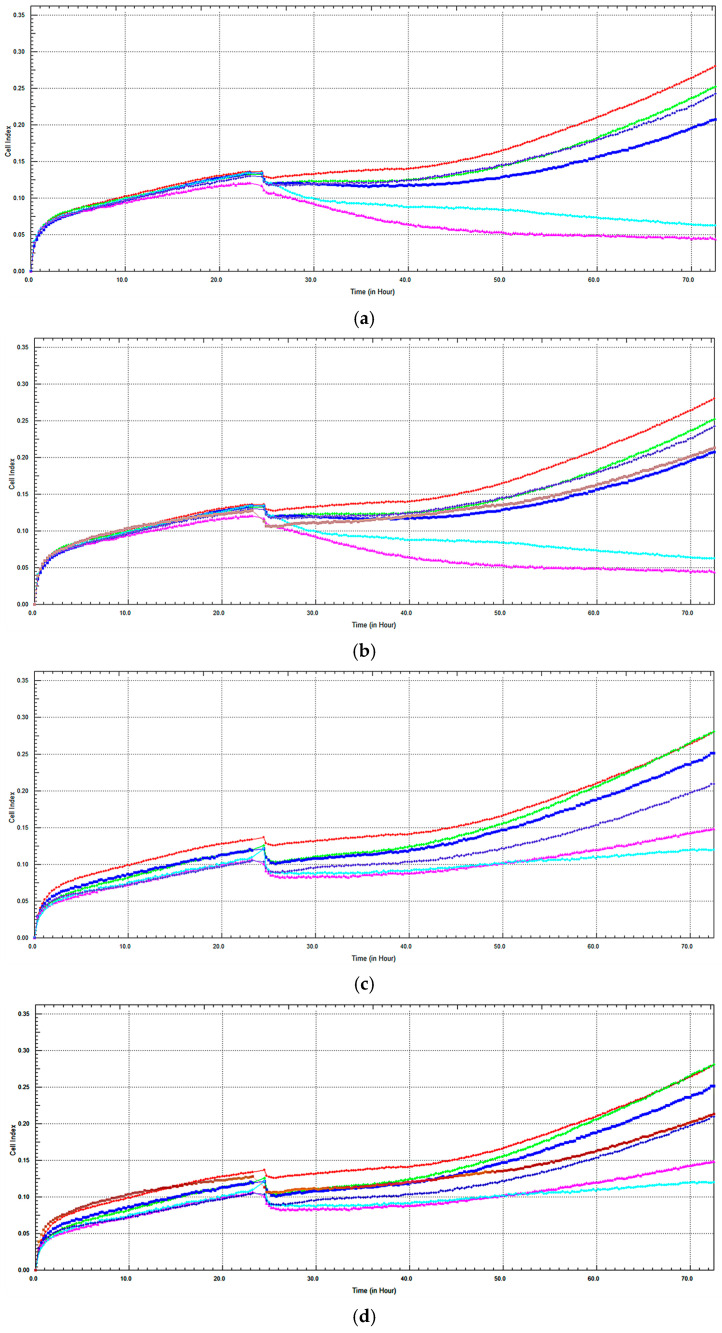
(**a**) Effect of CRD on RL95-2 after 48 h, based on time and concentration, evaluated using the xCELLigence RTCA system. (**b**) The effect of CRD extract and Dox on RL95-2 after 48 h, based on time and concentration, was evaluated using the xCELLigence RTCA system. (Red: Control, Green: 10 µg/mL, Purple: 30 µg/mL, Brown: Dox 3.37 µg/mL, Navy Blue: 100 µg/mL, Turquoise: 180 µg/mL, Pink: 240 µg/mL). (**c**) The time- and concentration-dependent effect of CRM extract on RL95-2 after 48 h, measured using the xCELLigence RTCA system. (**d**) The time- and concentration-dependent effect of CRM extract and Dox on RL95-2 after 48 h, measured using the xCELLigence RTCA system. (Red: Control, Green: 10 µg/mL, Navy blue: 30 µg/mL, Brown: Dox 3.37 µg/mL, Purple: 100 µg/mL, Pink: 180 µg/mL, Turquoise: 240 µg/mL) (CRD: *C. lycaonica* root dichloromethane extract, CRM: *C. lycaonica* root methanol extract, Dox: Doxorubicin).

To confirm the cell viability determined by the MTT assay and to assess real-time changes in cell viability, xCELLigence experiments were conducted with CRD, CRM, and Dox. The cell viability of CRD on RL95-2 after 48 h is shown in Figure 5a,b. CRD, which exhibited high cytotoxic activity at 180 and 240 µg/mL concentrations, showed a concentration-dependent decrease in cell viability compared to the control (Figure 5a). Figure 5b indicates that Dox reduced cell viability compared to the control. It was found that 180 and 240 µg/mL CRD concentrations induced higher cell death compared to Dox. CRD at a 100 µg/mL concentration exhibited cell viability similar to Dox.

The effect of CRM on cell viability in RL95-2 after 48 h is shown in Figure 5c. It was observed that CRM reduced cell viability in a concentration-dependent manner compared to the control at 48 h. High cytotoxic activity was observed at concentrations of 180 and 240 µg/mL, as evidenced by a decrease in cell index (CI). No reduction in cell viability was observed with 10 µg/mL CRM compared to the control. The effect of Dox on RL95-2 is presented in Figure 5d. Dox reduced the cell viability compared to the control. Similarly to the CRD concentrations, it was found that 180 and 240 µg/mL CRM concentrations induced higher cell death, and CRM at a concentration of 100 µg/mL exhibited a similar real-time cell profile to Dox.

IC_50_ values for 48 h were calculated as 98.78 µg/mL and 139.54 µg/mL for CRD and 60.02 µg/mL and 94.88 µg/mL for CRM, respectively, using MTT and xCELLigence assays. The results obtained indicated consistency between the two assays (Table 2).

### 2.4. Apoptotic Analysis of C. lycaonica Extracts

#### 2.4.1. Caspase 3 Assay

**Figure 6:**
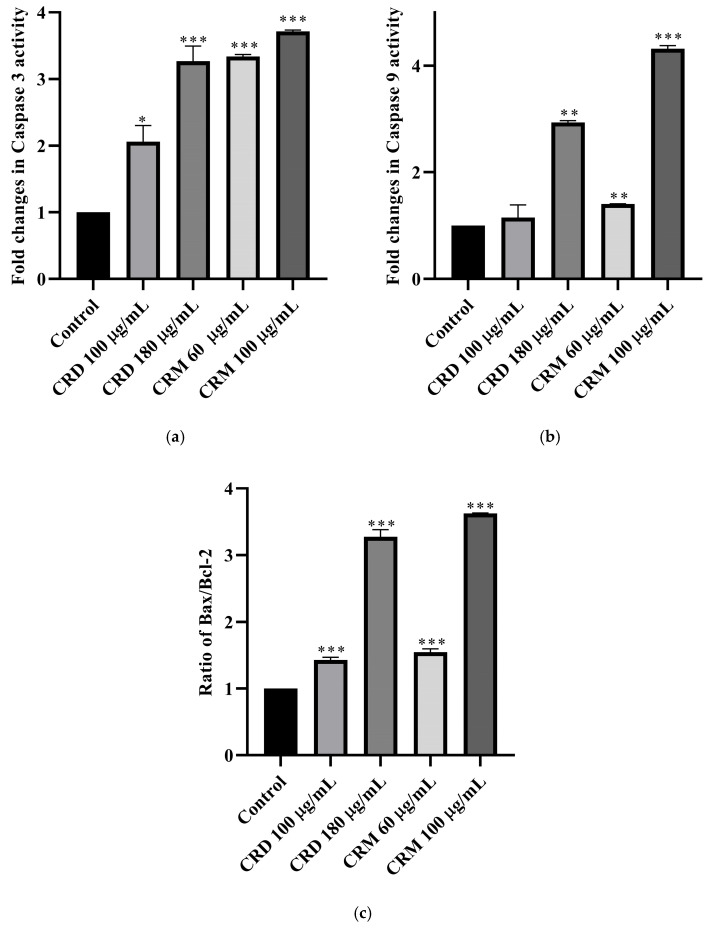
Fold changes in (**a**) caspase 3 and (**b**) caspase 9 activities (**c**) Bax/Bcl-2 ratio on RL95-2 for 48 h. (Data were analyzed using One-way ANOVA and post hoc Dunnett’s test (for comparison with the control group) in GraphPad Prism 8.3.0. All groups are presented as fold changes normalized to the untreated control. Results are presented as mean ± SD (*n* = 3). The *p*-value was <0.05. Significance levels: * < 0.05, ** < 0.01, *** < 0.001. CRD: *C. lycaonica* root dichloromethane extract, CRM: *C. lycaonica* root methanol extract).

The fold changes in caspase 3 activities of CRD and CRM on RL95-2 cells, compared to the control for 48 h, are shown in Figure 6a based on the ratio. At 100 and 180 µg/mL concentrations, CRD increased caspase 3 activity by 2.06 and 3.27 (*p* < 0.001) fold, respectively, compared to the control. At concentrations of 60 and 100 µg/mL, CRM increased caspase 3 activity by 3.57 (*p* < 0.001) and 3.62 (*p* < 0.001) fold, respectively (Table 3). Except for CRD 100 µg/mL, all concentrations significantly (*p* < 0.001) increased caspase 3 activity compared to the control, and this increase was found to be concentration-dependent. Among the extracts, a higher fold increase was observed in CRM.

#### 2.4.2. Caspase 9 Assay

The fold changes in caspase 9 activity of CRD and CRM compared to the control are shown in Figure 6b. At 100 and 180 µg/mL concentrations, CRD increased caspase 9 activity by 1.15 (*p* > 0.05) and 2.94 (*p* < 0.01) fold, respectively. In contrast, CRM increased caspase 9 activity by 1.41 (*p* < 0.01) and 4.32 (*p* < 0.001) fold at 60 and 100 µg/mL concentrations, respectively (Table 3). At CRM concentrations of 60 µg/mL (*p* < 0.01) and 100 µg/mL (*p* < 0.001), the fold increase was highly significant. A concentration-dependent fold increase was observed between these two concentrations. Among all extract concentrations, the highest fold increase in caspase 9 was observed in CRM 100 µg/mL.

#### 2.4.3. Bax/Bcl-2 Ratio

The ratio of the Bax/Bcl-2 activities obtained from the experiments was compared, and the changes relative to the control are shown in Figure 6c. The data showed a highly significant (*p* < 0.001) increase in all results. At 100 and 180 µg/mL concentrations, CRD increased the ratio by 1.43 (*p* < 0.001) and 3.27 (*p* < 0.001) times, respectively, compared to the control. In CRM, the ratio increased by 1.55 (*p* < 0.001) and 3.63 (*p* < 0.001) times at 60 and 100 µg/mL concentrations, respectively (Table 3). The increase observed in CRM was found to be higher compared to the other extract.

## 3. Discussion

Various anticancer studies on *Centaurea* species have focused on *C. polyclada* in ovarian cancer [13], *C. albonitens* in colon cancer [21], *C. kurdica* Reichardt, *C. virgata* Lam., C. saligna Wagenitz, and *C. fenzlii* in colorectal cancer [16,29], *C. jacea*, *C. calcitrapa*, *C. aegyptica*, and *C. mersinensis* in breast cancer [22,30,31], *C. aegyptica* and *C. nerimaniae* in cervical cancer [8,32]. However, to our knowledge, no cytotoxicity studies have been conducted on the RL95-2 endometrial cancer cell line using *C. lycaonica*. Therefore, identifying its potential anticancer effects and elucidating the underlying mechanisms constitute the primary focus of this study.

The species *Centaurea* is known for its rich secondary metabolites, making it an important cancer research target. Species of the genus contain sesquiterpene lactones [33], flavonoids [34], and various essential oils [35], all of which are believed to be responsible for their anticancer activity [36,37,38,39]. As previously identified by Fatullayev et al. (2023), *C. lycaonica* contained phenolic compounds and flavonoids responsible for antimicrobial and antioxidant activities [20]. This study explored the potential anticancer activity of *C. lycaonica*, and new data were added to the literature.

Several studies have used MTT and xCELLigence RTCA methods for cytotoxicity testing of *Centaurea* species. For example, Artun and Karagöz (2021) evaluated the methanol extract from *C. hermanni* leaves for cytotoxic and apoptotic effects on HeLa cells [40]. In their study, IC_50_ values from 48 h MTT and RTCA tests (15.74 µg/mL and 18.3 µg/mL, respectively) were consistent with our findings (CRD: 98.78 µg/mL and 139.54 µg/mL, and CRM: 60.02 µg/mL and 94.88 µg/mL). The positive control used in their study (Dox 4.57 µg/mL) also showed parallels with the concentration used in our study (3.37 µg/mL). However, differences in the cell lines, plant species, and plant parts used in the studies make direct comparisons difficult. The discrepancies in IC_50_ values between the two studies may arise due to these factors.

Additionally, Artun and Karagöz (2021) investigated the gene expression of caspases and found that caspase expression was increased [40]. Consistent with their findings, our study also observed a significant increase in caspase 3 and 9 activities compared to the control group. They also employed flow cytometry and gene expression analysis, which indicated increased apoptotic cell numbers and elevated expression of apoptosis-related genes. These findings suggest that different *Centaurea* species can activate various apoptotic pathways, showcasing the diversity in their anticancer mechanisms.

In a study conducted by Yağlıoğlu et al. (2014), the methanol extract of the root parts of *C. carduiformis* was used, similar to our research, and the IC_50_ values for African green monkey kidney (Vero) and HeLa cell lines were found to be 250 µg/mL and 735 µg/mL, respectively [41]. Although the differences in cell lines and testing methods may explain the variations in the results, these findings suggest that CRM, with its lower IC_50_ value (60 µg/mL), could be a stronger candidate for endometrial cancer.

Apoptosis is a complex process involving protein–protein interactions between pro-apoptotic and anti-apoptotic proteins. Most cancer therapies activate apoptotic pathways to overcome reduced apoptosis activity in cancer cells [42]. Caspase activation measurement is a standard method for detecting apoptosis. The Bcl-2 family, which includes pro-apoptotic (e.g., Bax) and anti-apoptotic (e.g., Bcl-2) proteins, is critical in apoptosis regulation. The Bax/Bcl-2 ratio is commonly used to assess apoptotic activity [4,5,6].

An example of studies on *Centaurea* species inducing cytotoxicity and activating apoptotic pathways through caspase activation and changes in the Bax/Bcl-2 ratio is a study conducted by Alper and Güneş (2019), in which it was found that the ethanol extracts of *C. solstitialis* increased caspase 3 activity by 1.65-fold compared to the control in the HeLa cell line [7]. In our study, CRD 180 µg/mL (3.27), CRM 60 µg/mL (3.57), and 100 µg/mL (3.62) induced a highly significant fold increase in caspase 3 activity compared to the control.

The study by Bahmani et al. (2018) on *C. albonitens* demonstrated caspase-dependent apoptosis with increased caspase 3 and Bax levels and decreased Bcl-2 levels [43]. Similarly, a study by Kayacan et al. (2018) on *C. nerimanie* found that its methanol extract increased apoptosis in HeLa and MDA-MB-231 (human breast cancer) cells through the caspase 3 pathway [8].

Similarly to our study, research involving methanol extract (Nasr et al., 2020) observed that *C. bruguierana* increased apoptosis and antiproliferation in the MCF-7 cell line, similar to CRD and CRM [9]. In that study, an increase in caspase 3 and 9 was observed, along with the upregulation of Bax and downregulation of Bcl-2, which was consistent with the findings of our study. Another study investigating the methanol extracts of *C. patula*, *C. pulchella*, and *C. tchihatchheffii* on the A375 (human melanoma cell line) revealed a decrease in anti-apoptotic Bcl-2 expression and an increase in pro-apoptotic Bax expression, confirming the pro-apoptotic effects [44].

Our study evaluated the effects of methanol and dichloromethane extracts of *C. lycaonica* at different concentrations. In a previous study by our research group, the dichloromethane and methanol extracts from the aerial parts of *C. lycaonica* demonstrated stronger apoptotic activity in HeLa cells via caspase and Bax/Bcl-2 pathways [27]. Evaluating different parts of the same plant on various cell lines is important for understanding their potential biological effects. When the findings of these two studies are compared, the methanol extract exhibited higher cytotoxic and apoptotic activity at lower doses. In our previous study, LC-HRMS analysis revealed that this apoptotic effect was likely due to the high diosmetin content. These results suggest that different parts of the same plant may have distinct phytochemical compositions.

Consistent with both our previous finding and the broader literature suggesting methanol may enhance the extraction of anticancer compounds [45,46], the current study on root extracts reaffirms the superior potency of the methanol extract. This was evidenced by a lower IC_50_ value, greater cytotoxicity at 100 µg/mL, and a more pronounced induction of apoptosis through caspase activation and Bax/Bcl-2 pathway modulation. 

Based on the findings from the LC-HRMS method, our study detected a high amount of hesperidin in the methanol extract. A study conducted by Cincin et al. (2018) found that hesperidin increased apoptosis by 1.5 times through the activation of caspase-3 in endometrial cancer [47]. As demonstrated in our study, the hesperidin compound is believed to be responsible for CRM’s high anticancer activity. Additionally, studies have shown that the diosmetin compound detected in CRD exhibits anticancer activity in ovarian [48], cervical, and breast cancer cells [49]. Our findings are parallel to the literature due to the pharmacological mechanism.

Our study provides the first investigation into the cytotoxic and apoptotic effects of *C. lycaonica* on the RL95-2 endometrial cancer cell line. The findings indicate that CRD and CRM possess significant cytotoxic and apoptotic effects, and, following the literature, apoptosis occurs in a caspase-dependent manner, as demonstrated by the increased activity of caspase 3 and 9 and the rise in the Bax/Bcl-2 ratio. Additionally, it highlights the importance of using methanol as a solvent in the extraction process due to CRM exhibiting higher apoptosis-inducing activity than CRD. However, a limitation of this study is the absence of cytotoxicity data in a non-cancerous, immortalized endometrial cell line, which is crucial for evaluating potential selective toxicity. Building directly upon these findings, future studies will prioritize investigating the selective cytotoxic effects of these extracts on normal endometrial cells (e.g., hTERT-immortalized) to assess their therapeutic potential and safety profile. Also, in the present study, quantification was performed using external calibration curves with excellent linearity (R^2^ > 0.995); however, no internal standards were applied. This methodological choice may be considered a limitation, as the inclusion of isotopically labeled or structurally similar internal standards would further improve the accuracy and precision of quantitative results.

## 4. Materials and Methods

### 4.1. Plant Material

The root material of *Centaurea lycaonica* was harvested in 2022 from the Seydişehir steppe (37°42′40″ N, 32°04′18″ E) in the Konya province in Turkey in August. The herbarium number is KNYA Herb. No: 30.214. The botanical identification of the plant material was made by Prof. Dr. Osman Tugay. The harvested material was dried in a shaded and well-ventilated environment.

### 4.2. Preparation of Plant Extract

In this study, the extraction was carried out using the maceration method. To obtain chemical constituents with different polarity profiles, sequential extraction was performed with solvents of increasing polarity, starting from less polar to more polar ones. A total of 40 g of powdered plant material was first macerated in 400 mL of dichloromethane at 36 °C in a shaking water bath for 24 h. The extract was then filtered, and the residual plant material was air-dried before being subjected to a second extraction with methanol [50]. The methanol extract was subsequently filtered and collected for further analysis. After the evaporation process to dryness under low pressure at 38 °C in a rotavapor, the obtained extracts were lyophilized and stored at −20 °C until use. Extraction efficiency: 10 mg extract/g dry weight for CRD extract and 48 mg extract/g dry weight for CRM extract.

### 4.3. RL95-2 Cell Line and Culture

The human endometrial carcinoma cell line RL95-2 (CRL-11506™) was obtained from the American Type Culture Collection (ATCC) and was provided by the Department of Pharmacology at Erciyes University, Türkiye. The medium used for the proliferation of RL95-2 cells was prepared with DMEM: F12 (Biological Industries, Cromwell, CT, USA, 01-170-1A) supplemented with 10% fetal bovine serum (FBS) (Biochrom, Cambridge, UK, S0115), 1% penicillin/streptomycin (Biological Industries 03-031-1C), and insulin (Sigma, St. Louis, MO, USA, I9278). The cells were regularly passaged and incubated at 37 °C in a standard cell culture atmosphere containing 5% CO_2_ and 95% humidified air. The prepared extracts were dissolved in DMSO to prepare a stock solution, which was diluted with FBS-free DMEM/F-12 culture medium immediately before each experiment. The final DMSO concentration in the medium was less than 0.1%. A stock solution of Dox was prepared in DMSO and then diluted with FBS-free DMEM/F-12 medium to achieve the working concentrations for treatments. The same DMSO concentration limit (0.1%) was applied for all treatments, including the control groups.

### 4.4. Cytotoxic Analysis

The cytotoxic effect of *C. lycaonica* extracts that is, CRD and CRM, on cell viability was first assessed using the MTT assay for general concentration screening. Subsequently, specific concentrations (for both CRD and CRM, concentrations of 10, 30, 100, 180, and 240 µg/mL) were determined from the initial screening and evaluated using the MTT assay and the xCELLigence RTCA Single Plate (SP) system (ACEA Biosciences Inc., San Diego, CA, USA).

#### 4.4.1. MTT Viability Assay

The cytotoxic effects of *C. lycaonica* root extracts on the RL95-2 cell line were evaluated using the MTT (Sigma-Aldrich, St. Louis, MO, USA, 475989) cell viability assay. RL95-2 cells were plated at a density of 10^4^ cells per well 24 h before treatment. Dox at a concentration of 3.37 µg/mL was used as a positive control [51]. The IC_50_ values, defined as the inhibitor concentration required to induce 50% cell death following 48 h of exposure, were determined for various concentrations of CRD and CRM derived from the dried root parts of *C. lycaonica*.

#### 4.4.2. xCELLigence RTCA System

RL95-2 cells were seeded into E-plates (Roche 05 232 368 001) at an appropriate density and allowed to adhere for 24 h in a 37 °C incubator with 5% CO_2_. The cells were treated with 10, 30, 100, 180, and 240 µg/mL CRD and CRM extracts. The positive control wells were treated with Dox (3.37 µg/mL). At the end of the 72 h monitoring period, impedance was calculated using the xCELLigence software, and the optimal concentration and time point for observed cytotoxicity were determined. The changes induced by the cells on impedance allow measurement, expressed as the CI value. Consequently, CI reflects the overall cell number, adhesion quality, and cell morphology, all of which can vary as a function of time [52].

#### 4.4.3. Apoptotic Analysis

The activity of caspase 3, 9, Bax, and Bcl-2 was assayed according to the manufacturer’s instructions (SunRed Biotechnology Co., Ltd., Shanghai, China, 20230721). RL95-2 cells were seeded into 6-well plates at a density of 1 × 10^6^ cells per well. The cells were then treated with various concentrations of CRD (100 and 180 µg/mL) and CRM (60 and 100 µg/mL) extracts and incubated for 48 h. The cells in the plate were washed twice with PBS (AppliChem, Darmstadt, Germany, A9177) and scraped using a scraper (Greiner, Kremsmünster, Austria, 541070). After centrifugation at 1000 rpm for 10 min at 4 °C (Biosan, Riga, Latvia, PST-60HL), the pellet was resuspended in cell lysis buffer with added protease inhibitors (A.G. Scientific, San Diego, CA, USA, B1352). The cells collected in the tubes were incubated on ice for 30 min, followed by sonication using a sonicator (Bandelin Sonopuls, Berlin, Germany, D-12207) to disrupt the cell membrane. After sonication, the cells were centrifuged at 1000 rpm for 10 min at 4 °C. The supernatant was collected for further analysis. The protein concentration in the obtained lysate was determined using the bicinchoninic acid (BCA) assay kit (Cell Signaling, Danvers, MA, USA, 7780).

#### 4.4.4. LC-HRMS Analysis

##### Qualitative Profile

The bioactive substances in the active extract were determined by full scan high-resolution accurate mass spectrometry (LC–HRMS). Analyses were performed using a DIONEX UltiMate 3000 RS pump, autosampler, and column oven-equipped LC system coupled to an Exactive Plus Orbitrap (Thermo Fisher Scientific, Waltham, MA, USA, ABD) high-resolution mass spectrometer with a heated electrospray ionization interface. The instrument operated in positive (Full MS/AIF) and negative (Full MS/AIF) modes. For chromatographic separation, a Phenomenex^®^ Gemini^®^ 3 μm NX-C18 110 Å (100 mm × 2 mm) column was used at 30 °C. The mobile phase consisted of 0.5% (*v/v*) acetic acid in water (A) and methanol (B) at a flow rate of 0.3 mL/min. 0–13 min, 0–98% B (*v*/*v*); 13–15 min, 98% B (*v*/*v*); 15–16 min, 98–0% B (*v*/*v*); and 16–20 min, 0% B (*v*/*v*). All gradients were linear. Prior to injection, the crude extract was filtered through a 0.22 µm PTFE syringe filter and diluted to 1 mg/mL in 50% methanol. Compounds were identified based on retention time, UV spectra, and high-resolution MS/MS fragmentation patterns, referencing commercial standards and literature data on previously reported molecules. Identified metabolites mainly belonged to phenolic acids, flavonoids, and tannins [53].

##### Quantitative Profile

The standard compounds for quantification were provided commercially. A series of standard solutions (10–1000 µg/mL) was prepared and injected in triplicate for the quantitative profile. Calibration curves were constructed for each standard compound by plotting peak area against concentration, showing excellent linearity (R^2^ > 0.995). Quantification of metabolites in the extract was carried out using these calibration curves, and concentrations of individual compounds were calculated from their corresponding linear regression equations.

#### 4.4.5. Statistical Analysis

All calculations from xCELLigence were obtained using the RTCA-integrated software of the xCELLigence system. Statistical analysis was performed by GraphPad Prism Software Version 8.3.0 (La Jolla, CA, USA) to compare differences in values between the control and experimental groups. The results are expressed as the mean ± SD calculated from 3 separate experiments. Statistically significant values were compared using one-way ANOVA and Dunnett’s post hoc test, and statistical significance was determined by *p* values < 0.05. Significance intervals were determined as * < 0.05; ** < 0.01 and *** < 0.001.

## 5. Conclusions

In conclusion, the present study showed that CRD and CRM were evaluated separately at different concentrations that inhibit the growth of human endometrial cancer cells RL95-2 by inducing apoptosis through the mitochondrial apoptotic pathway. A key finding was higher cytotoxicity of CRM at 100 µg/mL, which phytochemical analysis suggests is attributable to its higher hesperidin content. The differences between CRD and CRM regarding the biological activity of endometrial cancer cells have been identified based on phytochemical analysis. This study contributes to the literature on the molecular mechanisms of the root extracts of *C. lycaonica*. It encourages further advanced research on in vivo studies for their biological activities. *C. lycaonica* could be a potential candidate for containing valuable natural products against endometrial cancer cells. Diosmetin and hesperidin in the extracts highlight their potential importance in using the plant as a valuable pharmaceutical source.

## Figures and Tables

**Table 1:** The phytochemical composition of CRM and CRD extracts.

Compounds	RT (min)		[M − H]^−^ (*m*/*z*)	Content (µg/g Extract (dw))	
	CRM	CRD		CRM	CRM
4-Hydroxybenzoic acid	7.81	nd	137.02442	47.670 ± 0.125	nd ^1^
Salicylic acid	10.55	nd	137.02442	41.194 ± 0.852	nd
Syringic acid	8.9	8.9	197.04555	49.29 ± 1.243	41.598 ± 0.288
3,4-dihydroxybenzaldehyde	7.21	nd	137.02442	12.12 ± 0.218	nd
Vanillic acid	8.54	8.54	167.03498	219.43 ± 3.874	49.1 ± 0.510
Vanilin	8.97	8.98	151.04007	72.016 ± 1.842	177.19 ± 2.557
Coumaric acid	9.78	nd	163.04007	38.866 ± 0.973	nd
Caffeic acid	8.69	nd	179.03498	91.042 ± 2.184	nd
Ferulic acid	10.03	10.03	193.05063	147.148 ± 3.236	150.306 ± 1.388
Sinapic acid	10.15	nd	223.06120	21.426 ± 0.321	nd
Chlorogenic acid	8.21	nd	353.08781	2866.98 ± 28.124	nd
Quinic acid	0.85	0.85	191.05611	361.524 ± 7.231	9.52 ± 0.176
3-(4-Hydroxyphenyl) propionic acid	9.4	nd	165.05572	92.384 ± 1.847	nd
Apigenin	13.31	nd	269.04555	225.628 ± 4.512	nd
Apigenin 7-glucuronide	11.41	nd	445.07763	1317.994 ± 26.359	nd
Rutin	10.93	nd	609.14611	3639.834 ± 29.987	nd
Luteolin-7-O-glucuronide	10.84	10.86	461.07255	74.552 ± 1.491	19.492 ± 0.315
Diosmetin	13.46	13.45	299.05611	1095.524 ± 21.910	216.414 ± 2.498
Myricetin	nd	11.44	317.03029	nd	8.744 ± 0.227
Orientin	10.03	nd	447.09328	5.098 ± 0.051	nd
Afzelin	12.13	nd	431.09837	9.436 ± 0.141	nd
Hesperidin	11.45	nd	609.18249	48503.884 ± 29.995	nd
Ellagic acid	11.34	11.34	300.99899	25.312 ± 0.379	11.34 ± 0.229

^1^ nd: Not detected.

**Table 2:** IC_50_ values of Dox, CRD and CRM on RL95-2 were determined by MTT and the xCELLigence RTCA system for 48 h.

Method	Extract	IC_50_ (µg/mL)
MTT	CRD	98.78 ± 0.88
	CRM	60.02 ± 1.47
	Dox	3.37 ± 0.74
XCELLigence	CRD	139.54
	CRM	94.88

CRD: *C. lycaonica* root dichloromethane extract, CRM: *C. lycaonica* root methanol extract, IC_50_: Half maximal inhibitory concentration, MTT: (3-[4,5-dimethylthiazol-2-yl]-2,5 diphenyl tetrazolium bromide.

**Table 3:** Bax/Bcl-2 ratios and fold changes in caspase 3 and 9 activities on RL95-2.

Extract	Fold Changes in Caspase 3 Activity	Fold Changes in Caspase 9 Activity	Bax/Bcl-2 Ratio
CRD 100 µg/mL	+2.06	+1.15	+1.43
CRD 180 µg/mL	+3.27	+2.94	+3.27
CRM 60 µg/mL	+3.57	+1.41	+1.55
CRM 100 µg/mL	+3.62	+4.32	+3.63

CRD: *C. lycaonica* root dichloromethane extract, CRM: *C. lycaonica* root methanol extract.

## Data Availability

The original contributions presented in this study are included in the article. Further inquiries can be directed to the corresponding author.

## Supplementary Materials

The following supporting information can be downloaded at: https://www.mdpi.com/article/10.3390/ph18101558/s1, Figure S1: MS^n^ spectrum and Validation chromatograms (quantification peaks, confirming ions and ion overlays) and calibration graphs for hesperidin compound.; Figure S2: Calibration graphs for standard compounds.

## Abbreviations

The following abbreviations are used in this manuscript:

A375	Human melanoma cell line
BCA	Bicinchoninic acid
CRD	Dichloromethane extract from the root of *C. lycaonica*
CRM	Methanol extract from the root of *C. lycaonica*
CI	Cell Index
CTL	Cytotoxic T Lymphocytes
DMEM-F12	Dulbecco’s Modified Eagle Medium Nutrient Mixture F-12
Dox	Doxorubicin
FADD	Fas-associated death domain protein
FBS	Fetal Bovine Serum
HeLa	Cervical cancer
IC_50_	Half-maximal inhibitory concentration
MTT	3-[4,5-dimethylthiazol-2-yl]-2,5 diphenyl tetrazolium bromide
MCF-7	Breast cancer cell line
MDA-MB-231	Human breast cancer cell line
RL95-2	Human endometrial carcinoma
RTCA	Real-Time Cell Analyzer
Vero	African green monkey kidney cells
