# Supplementary material for: Phytochemical Profiling and Molecular Insights of Centaurea lycaonica: Apoptosis Induction via the Intrinsic Pathway in Endometrial Cancer Cells
Source: Pharmaceuticals (Basel). 2025 Oct 16;18(10):1558. doi: 10.3390/ph18101558 (PMC12566656; doi:10.3390/ph18101558)
Supplement: Supplementary file 1 [file pharmaceuticals-18-01558-s001.zip › pharmaceuticals-3869698-supplementary.pdf]

Phytochemical Profiling and Molecular Insights of *Centaurea lycaonica*: Apoptosis Induction via the Intrinsic Pathway in Endometrial Cancer Cells

Hesperidin

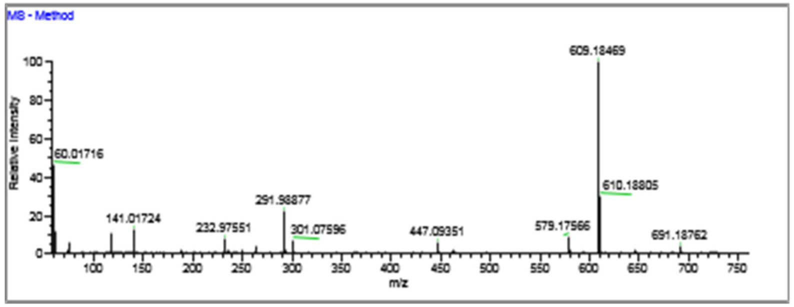

MS^n spectrum

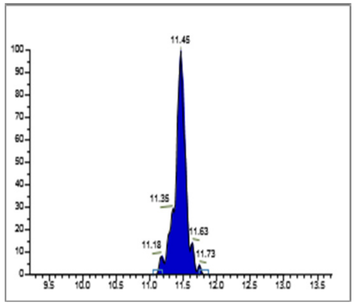

Quantification peak
m/z: 301.07596

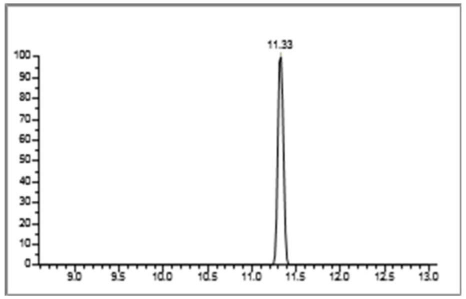

Confirming ion
m/z: 609.18249

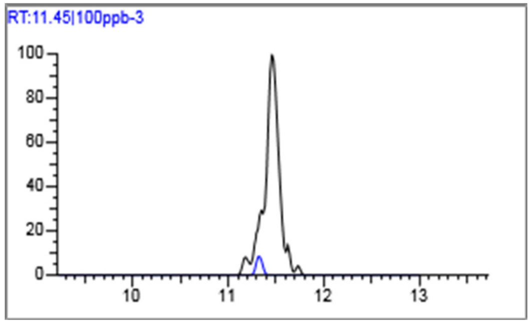

Ion overlay

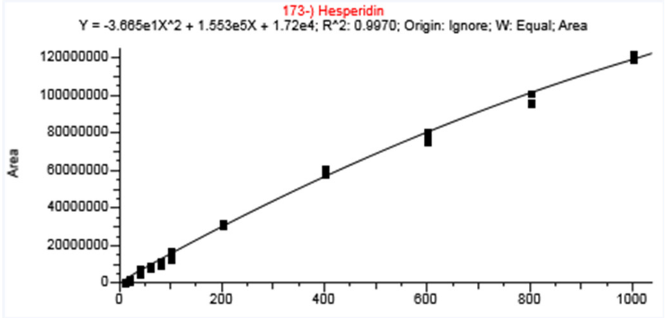

Calibration curve

Figure S1. MS^n spectrum and Validation chromatograms (quantification peaks, confirming ions and ion overlays) and calibration graphs for hesperidin compound.

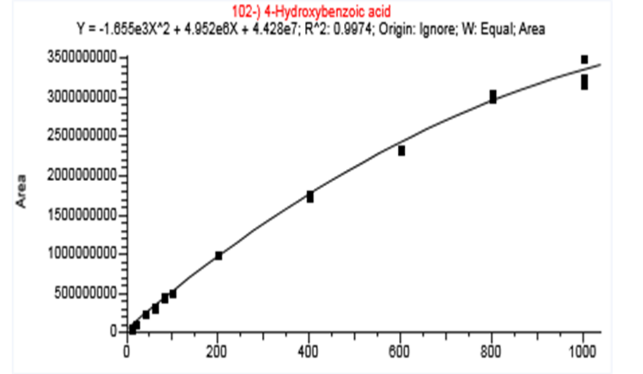
102-) 4-Hydroxybenzoic acid
Y = -1.655e3X^2 + 4.952e6X + 4.428e7; R^2: 0.9974; Origin: Ignore; W: Equal; Area
Area
3500000000
3000000000
2500000000
2000000000
1500000000
1000000000
500000000
0
0
200
400
600
800
1000

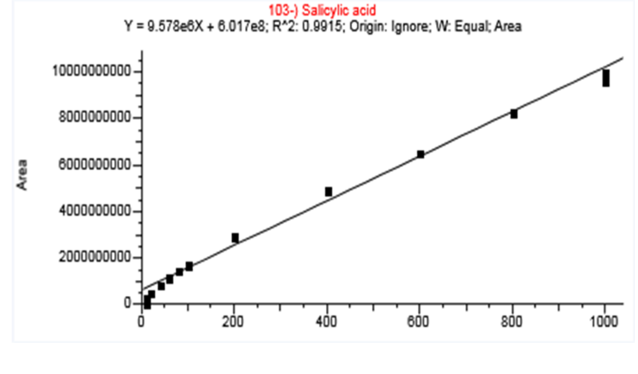
103-) Salicylic acid
Y = 9.578e6X + 6.017e8; R^2: 0.9915; Origin: Ignore; W: Equal; Area
Area
10000000000
8000000000
6000000000
4000000000
2000000000
0
0
200
400
600
800
1000

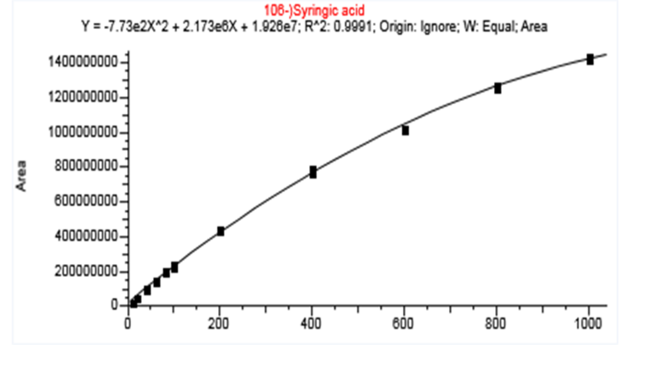
106-)Syringic acid
Y = -7.73e2X^2 + 2.173e6X + 1.926e7; R^2: 0.9991; Origin: Ignore; W: Equal; Area
Area
1400000000
1200000000
1000000000
800000000
600000000
400000000
200000000
0
0
200
400
600
800
1000

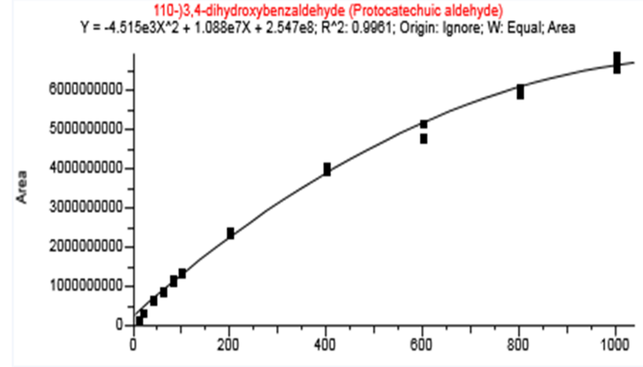
110-)3,4-dihydroxybenzaldehyde (Protocatechuic aldehyde)
Y = -4.515e3X^2 + 1.088e7X + 2.547e8; R^2: 0.9961; Origin: Ignore; W: Equal; Area
Area
6000000000
5000000000
4000000000
3000000000
2000000000
1000000000
0
0
200
400
600
800
1000

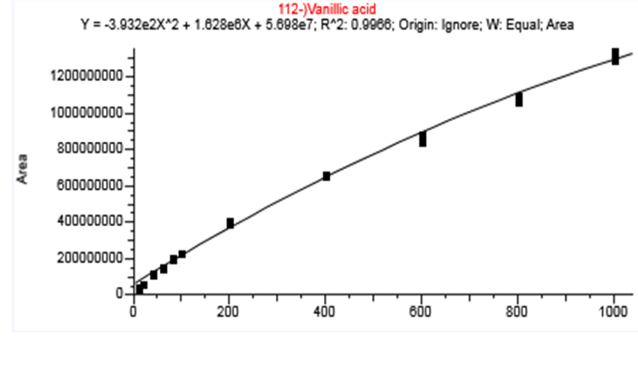
112-)Vanillic acid
Y = -3.932e2X^2 + 1.628e6X + 5.698e7; R^2: 0.9966; Origin: Ignore; W: Equal; Area
Area
1200000000
1000000000
800000000
600000000
400000000
200000000
0
0
200
400
600
800
1000

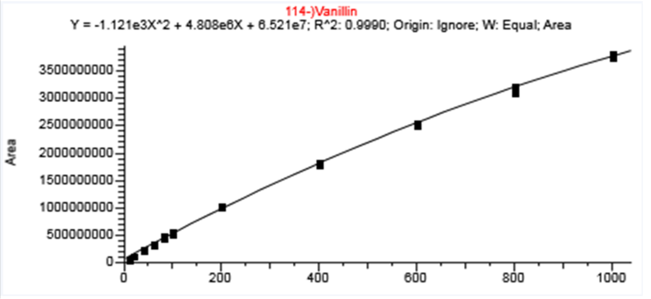
114-)Vanillin
Y = -1.121e3X^2 + 4.808e6X + 6.521e7; R^2: 0.9990; Origin: Ignore; W: Equal; Area
Area
3500000000
3000000000
2500000000
2000000000
1500000000
1000000000
500000000
0
0
200
400
600
800
1000

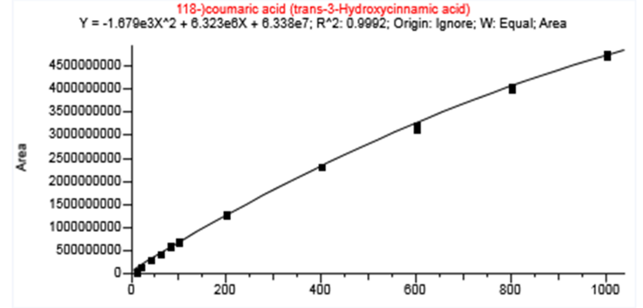
118-)coumaric acid (trans-3-Hydroxycinnamic acid)
Y = -1.679e3X^2 + 6.323e6X + 6.338e7; R^2: 0.9992; Origin: Ignore; W: Equal; Area
Area
4500000000
4000000000
3500000000
3000000000
2500000000
2000000000
1500000000
1000000000
500000000
0
0
200
400
600
800
1000

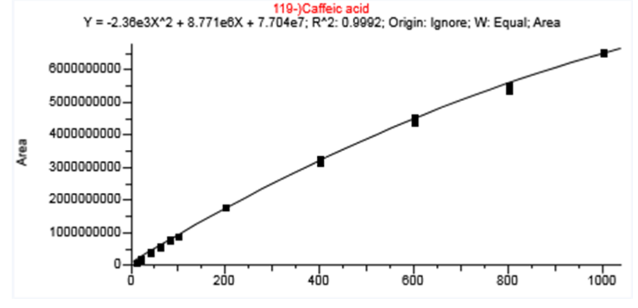
119-)Caffeic acid
Y = -2.36e3X^2 + 8.771e6X + 7.704e7; R^2: 0.9992; Origin: Ignore; W: Equal; Area
Area
6000000000
5000000000
4000000000
3000000000
2000000000
1000000000
0
0
200
400
600
800
1000

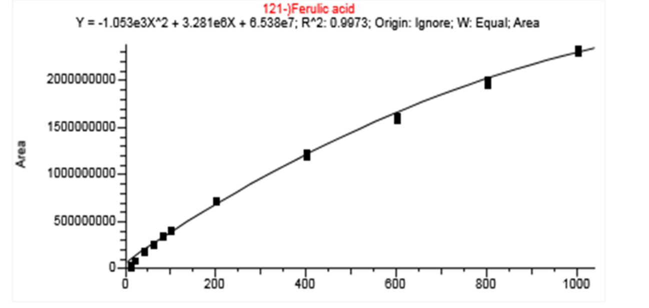
121-)Ferulic acid
Y = -1.053e3X^2 + 3.281e6X + 6.538e7; R^2: 0.9973; Origin: Ignore; W: Equal; Area
Area
2000000000
1500000000
1000000000
500000000
0
0
200
400
600
800
1000

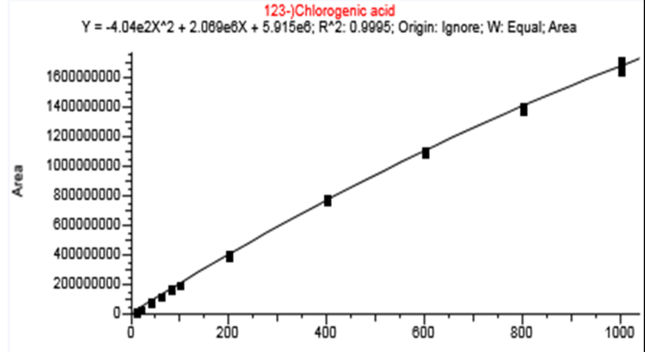
123-)Chlorogenic acid
Y = -4.04e2X^2 + 2.069e6X + 5.915e6; R^2: 0.9995; Origin: Ignore; W: Equal; Area
Area

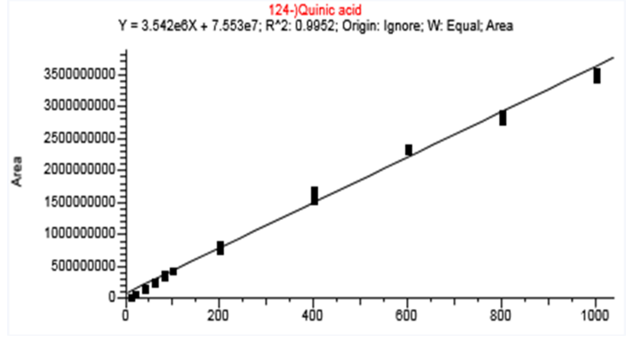
124-)Quinic acid
Y = 3.542e6X + 7.553e7; R^2: 0.9952; Origin: Ignore; W: Equal; Area
Area

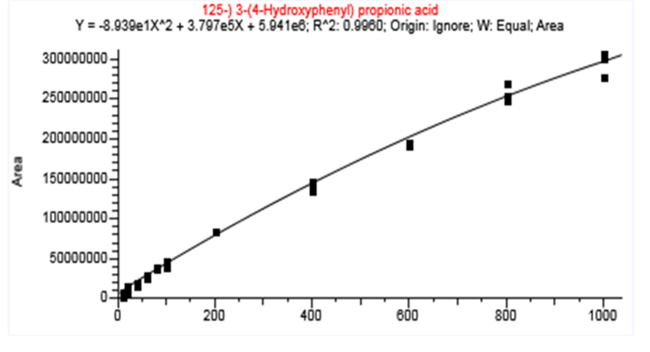
125-) 3-(4-Hydroxyphenyl) propionic acid
Y = -8.939e1X^2 + 3.797e5X + 5.941e6; R^2: 0.9960; Origin: Ignore; W: Equal; Area
Area

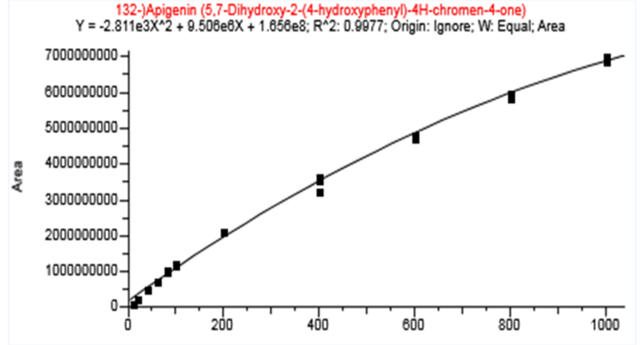
132-)Apigenin (5,7-Dihydroxy-2-(4-hydroxyphenyl)-4H-chromen-4-one)
Y = -2.811e3X^2 + 9.506e6X + 1.656e8; R^2: 0.9977; Origin: Ignore; W: Equal; Area
Area

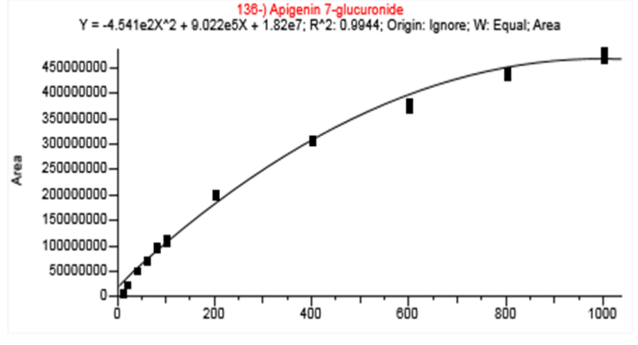
136-) Apigenin 7-glucuronide
Y = -4.541e2X^2 + 9.022e5X + 1.82e7; R^2: 0.9944; Origin: Ignore; W: Equal; Area
Area

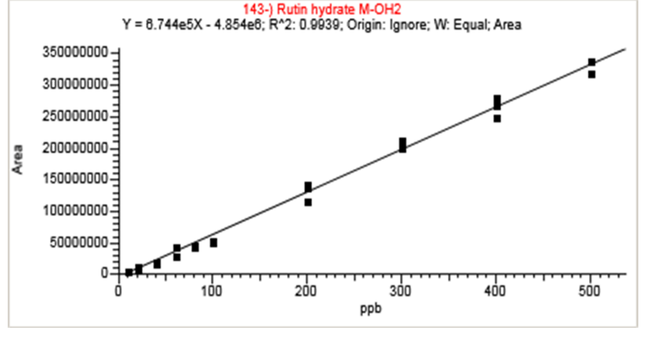
143-) Rutin hydrate M-OH2
Y = 6.744e5X - 4.854e6; R^2: 0.9939; Origin: Ignore; W: Equal; Area
Area
ppb

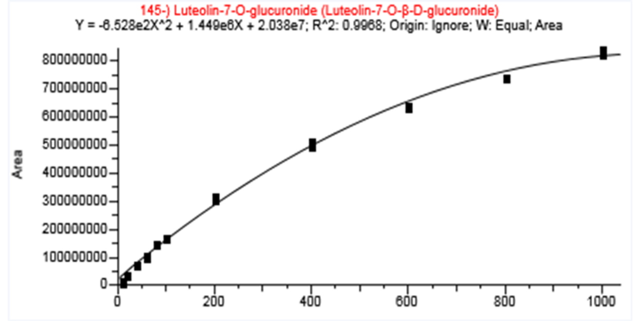
145-) Luteolin-7-O-glucuronide (Luteolin-7-O-β-D-glucuronide)
Y = -6.528e2X^2 + 1.449e6X + 2.038e7; R^2: 0.9968; Origin: Ignore; W: Equal; Area
Area

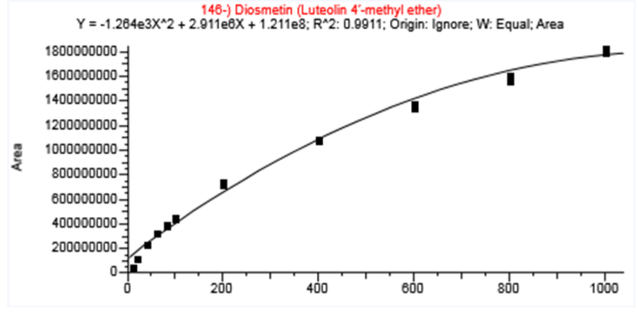
146-) Diosmetin (Luteolin 4′-methyl ether)
Y = -1.264e3X^2 + 2.911e6X + 1.211e8; R^2: 0.9911; Origin: Ignore; W: Equal; Area
Area

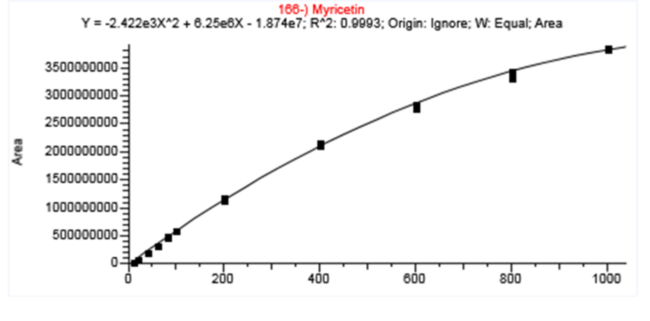
166-) Myricetin
Y = -2.422e3X^2 + 6.25e6X - 1.874e7; R^2: 0.9993; Origin: Ignore; W: Equal; Area
Area

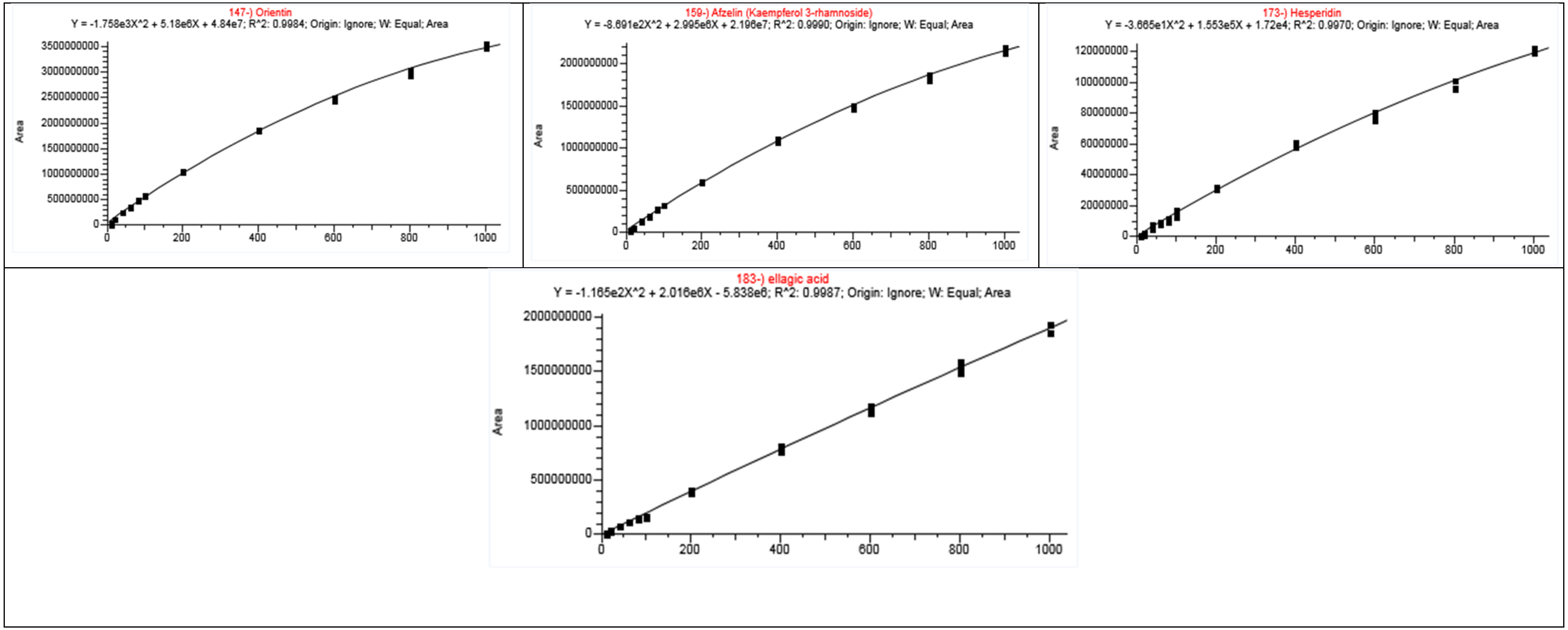

Figure S2. Calibration graphs for standard compounds.
